# TSG-6 inhibits IL-1β-induced inflammatory responses and extracellular matrix degradation in nucleus pulposus cells by activating the PI3K/Akt signaling pathway

**DOI:** 10.1186/s13018-022-03468-9

**Published:** 2022-12-28

**Authors:** Bing Wu, Xiaojin Guo, Xiujie Yan, Zikai Tian, Wei Jiang, Xin He

**Affiliations:** 1 Department of Orthopedics, Institute of the Third Medical Center, PLA (People’s Liberation Army) General Hospital, Beijing, 100039 China; 2 Department of Blood Transfusion, Institute of the Third Medical Center, PLA (People’s Liberation Army) General Hospital, Beijing, 100039 China; 3 Department of Anesthesiology, Institute of the Third Medical Center, PLA (People’s Liberation Army) General Hospital, Beijing, 100039 China

**Keywords:** Cervical disk degeneration (CDD), Tumor necrosis factor (TNF)-stimulated gene-6 (TSG-6), Inflammatory response, Extracellular matrix (ECM), PI3K/Akt signaling pathway

## Abstract

**Purpose:**

Tumor necrosis factor (TNF)-stimulated gene-6 (TSG-6), a secreted protein associated with inflammation, is believed to possess momentous and multiple anti-inflammatory and tissue-protective properties. However, the role and potential mechanism of TSG-6 in cervical disk degeneration (CDD) are still not clear. Hence, we aimed to explore the effect of TSG-6 on CDD.

**Methods:**

Quantitative reverse transcriptase polymerase chain reaction (qRT-PCR) or enzyme-linked immunosorbent assay was applied to detect the expression level of TSG-6 and IL-1β in normal and degenerated nucleus pulposus (NP) tissues. Then, qRT-PCR and western blot were adopted to test the TSG-6 protein expression after IL-1β treatment (10 ng/mL) in human NP cells (HNPCs). After over-expressing TSG-6, qRT-PCR was also utilized to evaluate the expression of TNF-α, IL-8, and IL-6 and the synthesis of sulfated glycosaminoglycans (sGAGs), western blot to check the expression of extracellular matrix (ECM) proteins [collagen II, aggrecan, and matrix metalloproteinase-3 (MMP-3)], pain-related molecules (CGRP, calcitonin gene-related peptide; NGF, nerve growth factor; SP, substance P), and PI3K/Akt signaling pathway-related proteins.

**Results:**

Briefly speaking, TSG-6 and IL-1β expression levels were significantly increased in CDD patient tissues; and IL-1β treatment could significantly increase TSG-6 expression in HNPCs. Further research revealed that, in addition to greatly promoting sGAGs synthesis, TSG-6 over-expression also inhibited TNF-α, IL-8, and IL-6 expression and ECM degradation in IL-1β-induced HNPCs. (The collagen II and aggrecan expression was up-regulated and MMP-3 expression was down-regulated.) Furthermore, over-expression of TSG-6 could decrease the levels of CGRP, NGF, and SP protein expression and activate the PI3K/Akt signaling pathway in IL-1β-treated HNPCs.

**Conclusion:**

TSG-6 inhibits inflammatory responses, ECM degradation, and expression of pain-related molecules in IL-1β-induced HNPCs by activating the PI3K/Akt signaling pathway.

## Introduction

Cervical spondylosis, also known as cervical vertebra syndrome, is a degenerative disease that mainly occurs in the elderly [1]. The latest survey shows that, due to the continuously accelerating pace of life and prolonged life expectancy, cervical spondylosis is increasingly prevalent and serious, and the patients also tend to be younger [2, 3]. Undoubtedly, cervical spondylosis increases the social and economic burden. Generally, cervical spondylosis is caused by cervical disk degeneration (CDD) and cervical hyperosteogeny [1]. CDD is a common degenerative spine disease. Patients at the onset suffer from neck and shoulder pain, which can spread to the head or upper limbs. Notably, serious CDD may induce lower limb spasticity, making patients difficult to walk. Additionally, CDD also declines patients’ quality of life due to symptoms such as neck and back pain, upper and lower limb weakness, and vertigo [4]. Currently, comprehensive clinical treatments are applied to most patients with CDD. Among these treatments, surgery and exercise are the most common choices [3]. However, surgical treatment is expensive and exercise therapy requires enough free time for rehabilitation. Comprehensive treatments are difficult for the bulk of patients with CDD due to limitations such as time, money, long working hours, and unhealthy lifestyles. Moreover, the pathogenesis of CDD is still poorly understood. Therefore, elucidating the pathogenesis of CDD is extremely important for the development of precise targeted therapy.

The intervertebral disk (IVD) is composed of three parts: superior endplate, central nucleus pulposus (NP), and external annulus fibrosus (AF) [5]. NP cells (NPCs) in NP tissues play critical roles in maintaining IVD integrity by producing extracellular matrix (ECM) components (including proteoglycans and collagen II) and secreting cytokines [6]. Additionally, a study has reported that abnormal functions of NP cells, such as abnormal cell proliferation, apoptosis, senescence, inflammation, and ECM synthesis and degradation, are involved in the progression of CDD [7]. Therefore, finding effective methods to maintain the normal inflammatory response and ECM synthesis in NP cells is of great importance for treating CDD.

As research has deepened, multiple key genes have been discovered to play an important role in CDD. For instance, Yu et al. [8] claimed that ubiquitin-specific protease 15 (USP15) promoted apoptosis in degenerative NPCs by inhibiting the PI3K/AKT signaling pathway. Lu et al. [9] stated that miRNA-424-5p regulated cell apoptosis and proliferation by targeting Bcl2 in NPCs. Overall, the occurrence of CDD is closely associated with abnormal gene expression. However, it cannot be ignored that a large number of potentially undiscovered key genes also affect CDD. Therefore, further exploration is needed to reveal the mechanism of functional imbalance in NP cells. Notably, tumor necrosis factor (TNF)-stimulated gene-6 (TSG-6) is an inflammation-related secreted protein that takes on important and multiple tissue-protective and anti-inflammatory properties [10]. Besides, based on previous studies, TSG-6 is correlated with a variety of diseases and shows beneficial effects in the treatment of many disease models, including a significant up-regulation of TSG-6 expression after spinal cord injury [11]. Moreover, the correlation of TSG-6 with human arthritis has been demonstrated [12]. It is worth noting that Yang et al. [13] observed that bone marrow mesenchymal stem cells could secret TSG-6 to effectively relieve neuropathic pain after they were injected into the cerebrospinal fluid of chronic constriction injury (CCI) rats. All in all, TSG-6 shows a significant effect in treating excessive inflammation and relieving pain. However, none of the studies have revealed the effect of TSG-6 on CDD. Therefore, in this study, we tried to further investigate the effects and mechanisms of TSG-6 on CDD through analysis of TSG-6 expression in CDD patient tissues and in vitro experiments. On the basis of this paper, we provided new insights into the treatment of CDD.

## Materials and methods

### Tissue sample collection

Degenerative NP tissues were collected from CDD patients (DNP group, *n* = 20), and normal NP tissues of IVD were obtained from cervical trauma patients (NNP group, *n* = 20). By the way, all of the selected patients received orthopedic surgery. Besides, patients agreed with the tissue sample collection and signed the informed consent forms. All study procedures were approved by the ethics committee of the Institute of the Third Medical Center, PLA (People's Liberation Army) General Hospital.

### Cell culture and treatment

Human NPCs (HNPCs) were purchased from ScienCell (Carlsbad, CA, USA). They were placed in Dulbecco's modified eagles medium (DMEM) containing 10% of fetal bovine serum and cultured in an incubator with 5% of CO_2_ at 37 °C.

Subsequently, through Lipo3000 transfection kit (Thermo Fisher Scientific, Inc., USA), pcDNA3.2-TSG-6 plasmids (TSG-6 OE) or empty plasmid (NC) was transfected into HNPCs to over-express TSG-6. After 6 h of transfection, the medium was replaced by a fresh one. Upon another 48 h of culture, the cells were collected for the following research.

The cells were divided into Control (CTRL) group (normally cultured HNPCs), IL-1β group [HNPCs with 48 h of IL-1β (10 ng/mL) treatment], TSG-6 group (HNPCs with over-expressed TSG-6), and TSG-6 + IL-1β group (IL-1β-treated TSG-6-over-expressing HNPC).

### Real-time quantitative reverse transcriptase polymerase chain reaction (qRT-PCR) analysis

The total RNA was extracted from cells and tissues using Total RNA Extraction Kits (Thermo Fisher Scientific, Inc.) in light of the instruction. Later, a NanoDrop One ultramicrospectrophotometer (Thermo Fisher Scientific, Inc.) was applied to measure the concentration and purity of the extracted RNA. Subsequently, cDNA was synthesized using the PrimeScript RT Kit (Takara, Japan). Next, qRT-PCR was performed to evaluate transcriptional levels of TSG-6, IL-1β, TNF-α, IL-8, and IL-6 according to the TB Green® Premix Ex Taq™ II (Tli RNase H Plus) kit instructions. GAPDH served as a housekeeping gene, and the relative expression of the target gene was calculated via the 2^−ΔΔCt^ method. The primer sequences are listed in Table 1.

**Table 1:** Quantitative primers

Genes	Sequences(5′–3′)
TSG-6	F: 5′- TCACCTACGCAGAAGCTAAGGC- 3′
	R: 5′- TCCAACTCTGCCCTTAGCCATC- 3′
IL-1β	F: 5′- CCACAGACCTTCCAGGAGAATG- 3′
	R: 5′- GTGCAGTTCAGTGATCGTACAGG- 3′
TNF-α	F: 5′- CTCTTCTGCCTGCTGCACTTTG- 3′
	R: 5′- ATGGGCTACAGGCTTGTCACTC- 3′
IL-6	F: 5′- AGACAGCCACTCACCTCTTCAG- 3′
	R: 5′- TTCTGCCAGTGCCTCTTTGCTG- 3′
IL-8	F: 5′- GAGAGTGATTGAGAGTGGACCAC- 3′
	R: 5′- CACAACCCTCTGCACCCAGTTT- 3′
GAPDH	F: 5′- GTCTCCTCTGACTTCAACAGCG- 3′
	R: 5′- ACCACCCTGTTGCTGTAGCCAA- 3′

### Enzyme-linked immunosorbent assay (ELISA)

The degenerative and normal NP tissues were lysed by RIPA lysate (Beyotime, China) and then centrifuged at 4 °C. The supernatant was used to detect the contents of IL-1β by ELISA Detection Kits (Beyotime, China) following the manufacturer's instructions.

### Western blot

Total protein from cells was extracted using RIPA lysate (Beyotime, China), followed by the detection of the protein concentration using BCA kits (Beyotime, China). Next, the protein (25 μg) was separated by sodium dodecyl sulfate–polyacrylamide gel electrophoresis (SDS-PAGE) and then transferred to polyvinylidene fluoride membranes. Later, the membranes were blocked with 5% skimmed milk for 1 h at ambient temperature. Then, they were incubated with primary antibodies TSG-6 (ab267469, 1:1000), Collagen II (ab6586, 1:1000), Aggrecan (ab3778, 1:1000), metalloproteinase-3 (MMP-3, ab285407, 1:1000), calcitonin gene-related peptide (CGRP, ab283568, 1:1000), nerve growth factor (NGF, ab52987, 1:1000), substance P (SP, ab220422, 1:1000), PI3K (ab278545, 1:1000), AKT (ab8805, 1:1000), p-PI3K (ab140307, 1:1000), and p-AKT (ab285140, 1:1000) overnight at 4 °C. All antibodies were purchased from Abcam, Cambridge, UK. Subsequently, the mixture was rinsed three times with TBST; then, another 1 h of incubation at ambient temperature was performed with horseradish peroxidase-conjugated goat anti-mouse IgG secondary antibody (ab205719, 1:5000) and goat anti-rabbit IgG antibody (ab6721, 1:5000). Next, immunoreactive bands were visualized using enhanced chemiluminescence reagents (Thermo Fisher Scientific, Waltham, USA) and imaged by a ChemiDoc XRS Plus Luminescent Image Analyzer (Bio-Rad). Finally, the optical density of the band intensities was determined using Image-Pro Plus 6.0 software, and relative expression levels of target proteins were normalized to band intensities of β-actin (ab8226, 1:1000).

### Generation of sulfated glycosaminoglycans (sGAGs)

The supernatant was collected to detect the content of sulfated glycosaminoglycans (sGAGs) in the cells. More specifically, the sGAGs’ content was checked using Blyscan™ sGAG Isolation & Concentration Pack Kits (AmyJet Scientific Inc.).

### Statistics and analysis

SPSS 26.0 software was adopted for statistical analysis of the collected data in this paper. Comparisons between two groups were made by T test, and the comparison among multiple groups by one-way analysis of variance. Outcomes were presented as mean ± standard deviation (SD), and *P* < 0.05 was considered statistically different.

## Results

### TSG-6 and IL-1β express highly in the tissues of patients with cervical disk degeneration (CDD)

**Fig. 1:**
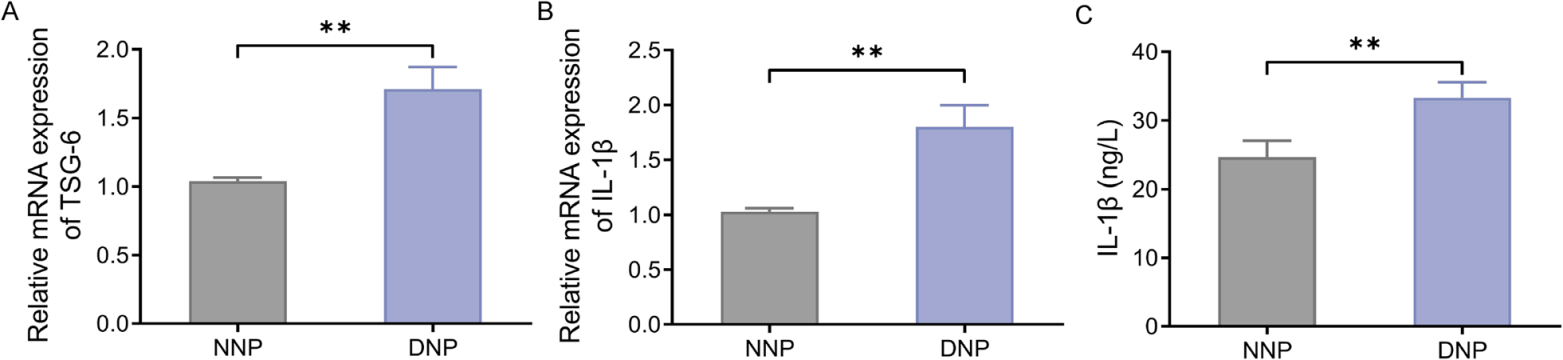
High TSG-6 and IL-1β expression in degenerative nucleus pulposus tissue of intervertebral disk. **A**–**B**, qRT-PCR to measure the expression of TSG-6 and IL-1βin normal/degenerative intervertebral disk nucleus pulposus tissues; **C**, ELISA to measure the concentration of IL-1β in normal/degenerative intervertebral disk nucleus pulposus tissues, *n* = 20, **P < 0.01 versus IVD group

Firstly, qRT-PCR was conducted to ensure TSG-6 and IL-1β expression in CDD patient tissues. Briefly speaking, compared with those in the IVD group, the expression levels of TSG-6 and IL-1β in the CDD group were significantly increased (Fig. 1A, B). The ELISA result also showed that the concentration of IL-1β in CDD patient tissues was significantly higher than that in normal tissues (Fig. 1C). In other words, TSG-6 and IL-1β levels were abnormally high expressed in CDD patient tissues.

### High TSG-6 expression in IL-1β-induced human nucleus pulposus cells

**Fig. 2:**
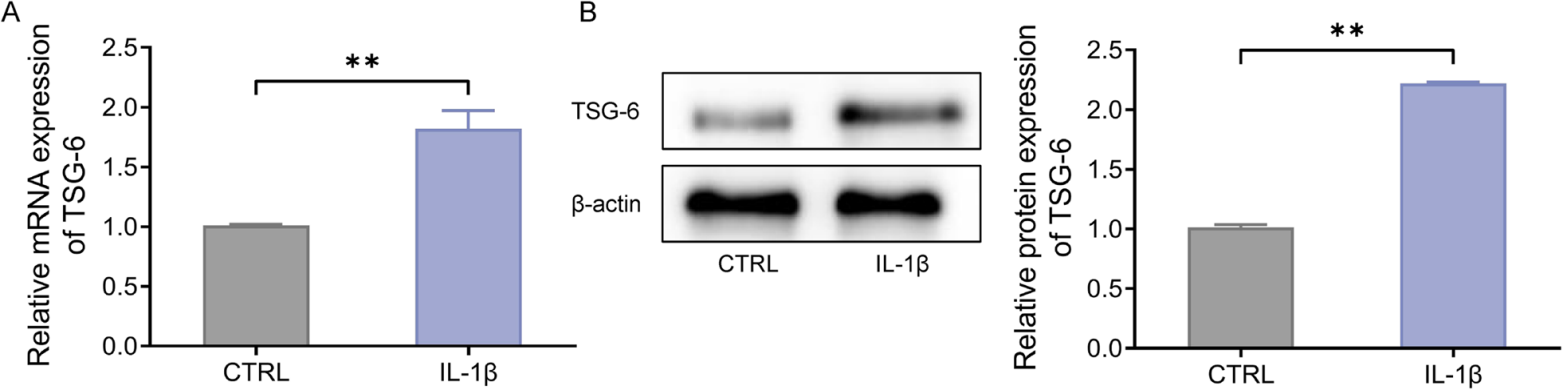
Highly expressed TSG-6 in IL-1β-treated human nucleus pulposus cells. **A**, TSG-6 mRNA levels in IL-1β-induced human nucleus pulposus cells were checked by qRT-PCR; **B**, TSG-6 protein expression levels in IL-1β-induced human nucleus pulposus cells were checked by western blot, respectively. **P < 0.01 versus CTRL group

To reveal the role of TSG-6 and IL-1β in CDD, IL-1β was utilized to induce an inflammatory cell model of HNPC. The outcomes of qRT-PCR and western blot presented that the mRNA and protein expression levels of TSG-6 were notably up-regulated in the IL-1β group compared with those in the CTRL group (Fig. 2A, B). Therefore, we reckoned that IL-1β-induced inflammatory responses promoted TSG-6 expression.

### Over-expression of TSG-6 attenuates IL-1β-induced degradation of extracellular matrix in human nucleus pulposus cells

**Fig. 3:**
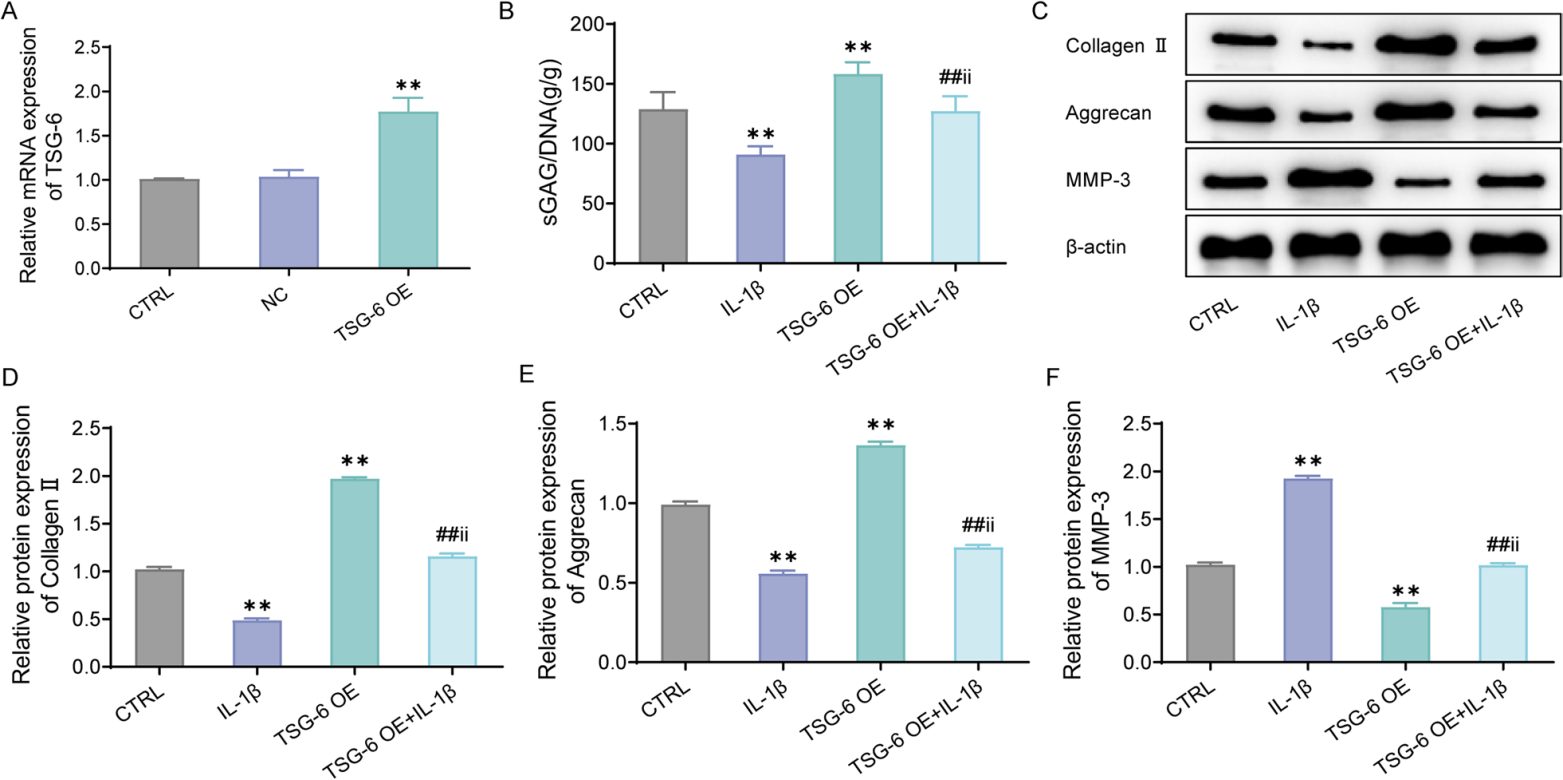
Over-expression of TSG-6 attenuates IL-1β-caused extracellular matrix degradation in human nucleus pulposus cells. **A**, TSG-6 mRNA expression levels after over-expression of TSG-6 in human nucleus pulposus cells were checked by qRT-PCR; **B**, related kits to detect the generation of sGAGs in each group of cells; **C**–**E**, western blot to test collagen II, aggrecan and MMP-3 protein expression in the cells. **P < 0.01, versus CTRL group; ##*P* < 0.01, versus IL-1β group; ii *P* < 0.01, versus TSG-6 OE group

To investigate the effect of TSG-6 on IL-1β-induced ECM, IL-1β was employed to stimulate HNPC that over-expressed TSG-6 in this study. First, qPCR was conducted to confirm the over-expression of TSG-6 after transfection. The results showed that the expression of TSG-6 was significantly increased compared to the CTRL group, which indicated that TSG-6 OE was successfully established (Fig. 3A). Relative to the CTRL group, the IL-1β group presented greatly suppressed levels of sGAGs, while the TSG-6 OE group showed significantly up-regulated levels of sGAGs in HNPCs (Fig. 3B). Subsequently, based on the western blot results, IL-1β significantly decreased collagen II and aggrecan protein expression, while increased MMP-3 protein expression; over-expression of TSG-6 greatly increased collagen II and aggrecan protein expression, while decreased MMP-3 expression in IL-1β-induced HNPC (Fig. 3C–E). Overall, TSG-6 could ameliorate ECM degradation and promote sGAGs’ generation in IL-1β-induced HNPC.

### Over-expression of TSG-6 decreases IL-1β-induced inflammatory factor expression in human nucleus pulposus cells

**Fig. 4:**
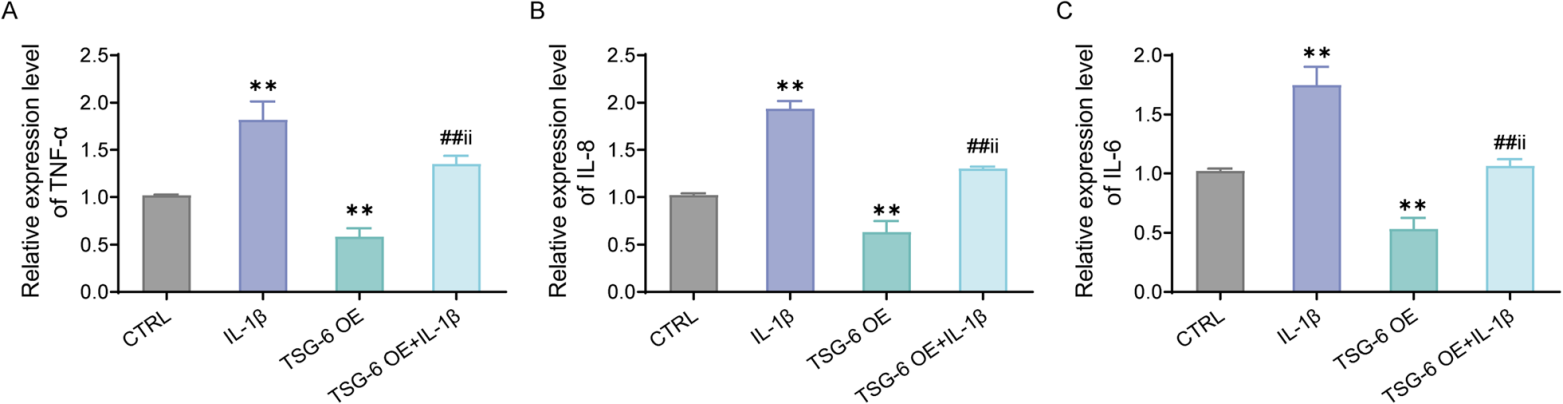
Over-expression of TSG-6 declines the inflammatory factor expression in human nucleus pulposus cells treated by IL-1β. **C**, The expression levels of TNF-α (**A**), IL-8 (**B**) and IL-6 (**C**) in the cells by qRT-PCR, **P < 0.01, versus CTRL group; ##*P* < 0.01, versus IL-1β group; ii *P* < 0.01, versus TSG-6 OE group

To explore the effect of over-expression of TSG-6 on IL-1β-induced inflammatory responses, HNPCs were treated with IL-1β for 48 h. Next, qRT-PCR was applied to measure the inflammatory factor expression in each group. Shortly speaking, compared with the CTRL group, IL-1β could greatly promote TNF-α, IL-8, and IL-6 expression in HNPCs, while TSG-6 over-expression significantly inhibited IL-1β-induced inflammatory factor secretion in the cells (Fig. 4A–C). On the basis of the above findings, over-expression of TSG-6 could suppress IL-1β-induced inflammatory responses in HNPCs.

### Over-expression of TSG-6 inhibits IL-1β-induced expression of pain-related molecules in human nucleus pulposus cells

**Fig. 5:**
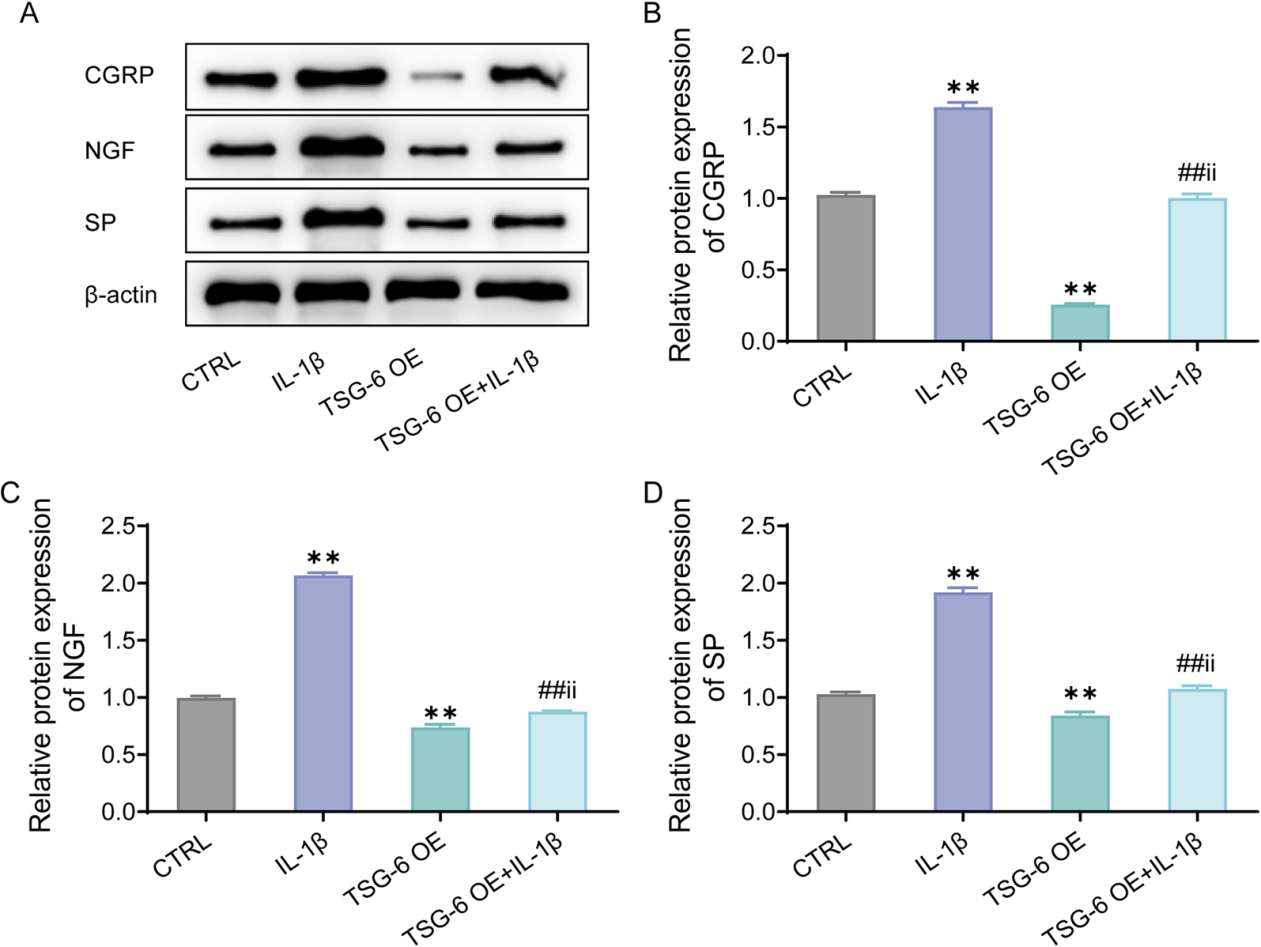
Over-expression of TSG-6 suppresses IL-1β-induced expression of pain-related molecules in human nucleus pulposus cells. **A**–**D**, Western blot to detect the expression of CGRP (**B**), NGF (**C**) and SP (**D**) in the cells, **P < 0.01, versus CTRL group; ##*P* < 0.01, versus IL-1β group; ii *P* < 0.01, versus TSG-6 OE group. CGRP, calcitonin gene-related peptide; NGF, nerve growth factor; SP, substance P

Next, the expression of pain-related molecules in HNPCs was detected by western blot. The detection outcomes displayed that, in comparison with the CTRL group, the expression of CGRP, NGF, and SP proteins was notably increased in the cells after IL-1β treatment; while over-expression of TSG-6 could obviously inhibit the above expression in IL-1β-induced HNPCs (Fig. 5A–D). In a nutshell, TSG-6 over-expression inhibited IL-1β-induced expression of pain-related molecules in HNPCs.

### Over-expression of TSG-6 activates the PI3K/Akt signaling pathway in IL-1β-induced human nucleus pulposus cells

**Fig. 6:**
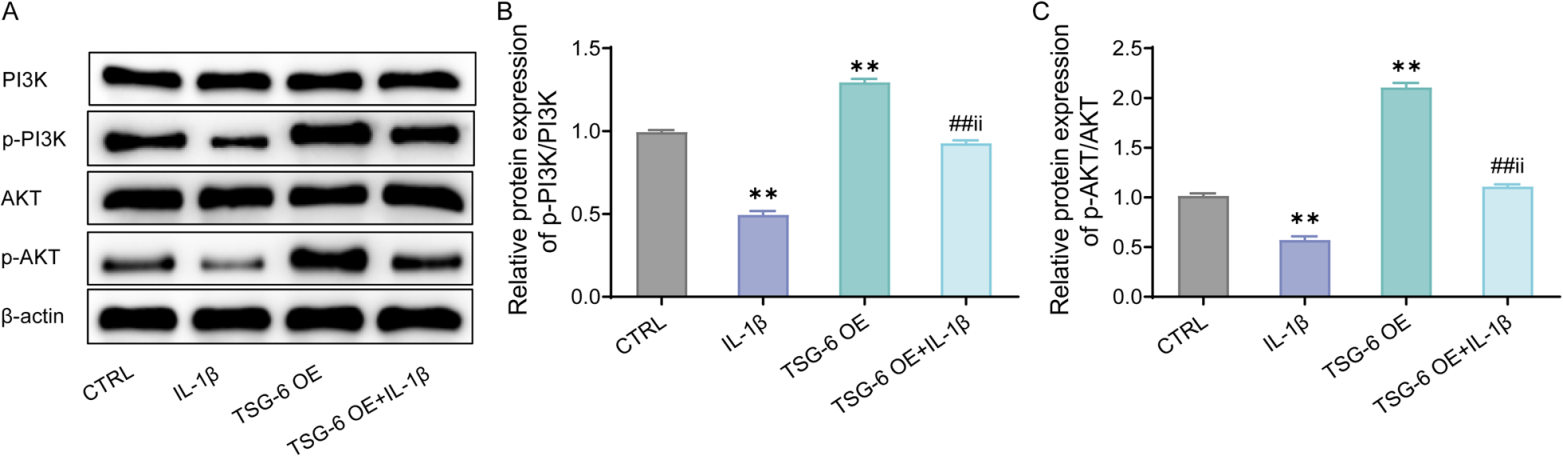
Activation of the PI3K/Akt signaling pathway by TSG-6 in IL-1β-induced human nucleus pulposus cells. **A**–**C**, Western blot to determine the expression levels of PI3K/Akt signaling pathway-related proteins (PI3K, AKT, p-PI3K and p-AKT) in the cells. **P < 0.01, versus CTRL group; ##*P* < 0.01, versus IL-1β group; ii *P* < 0.01, versus TSG-6 OE group

To further discuss the mechanism of TSG-6 in CDD, the PI3K/Akt signaling pathway in IL-1β-treated HNPC was tested by western blot in this study. To be specific, when making a comparison with the CTRL group, the p-PI3K and p-AKT expression levels in HNPCs were markedly decreased after IL-1β treatment, while over-expression of TSG-6 could greatly increase the above two expression levels (Fig. 6A–C). Consequently, TSG-6 may improve the inflammatory response and pain sensation in CDD patients by activating the PI3K/Akt signaling pathway.

## Discussion

Pain and mobility loss caused by CDD seriously affect the physical and mental health of patients. Currently, neither therapeutic regimens nor treatment results are satisfactory due to the unclear pathogenesis of CDD. A previous study showed the great potential of TSG-6 in the treatment of spinal diseases [14]. In this research, TSG-6 and IL-1β were abnormally up-regulated in tissues from CDD patients, and over-expression of TSG-6 in HNPCs could significantly ameliorate IL-1β-induced inflammatory response, synthesis and degradation of ECM, and expression of pain-related molecules. Also, further tests revealed that the mechanism by which TSG-6 treats CDD may be achieved through activation of the PI3K/Akt signaling pathway.

Pro-inflammatory cytokines have been reported as key factors in inducing pain in CDD patients, and among them, IL-1β is considered the most important cytokine involved in various pathological processes in CDD [15]. Shen et al. [16] pointed out that IL-1β promoted degenerative HNPC apoptosis by activating its downstream signaling target (NF-κB). Besides, IL-1β also induces apoptosis and autophagy via the mitochondria pathway in degenerative HNPCs [17]. Furthermore, under IL-1β stimulation, TSG-6 expression with significant anti-inflammatory effects is raised and plays a role in cell or tissue protection (including osteoclasts, monocytes, and macrophages) [18]. Interestingly, simultaneous abnormal up-regulation of IL-1β and TSG-6 expression was observed by detecting clinical NP tissue samples of CDD patients in this paper. Therefore, an excessive inflammatory response in CDD patients may stimulate NPCs to produce large amounts of TSG-6 for self-protection.

To further verify the outcomes in our paper, HNPCs transfected with over-expressed TSG-6 were stimulated by IL-1β. Specifically, the generation of sGAGs was significantly reduced in IL-1β-induced HNPCs, while over-expression of TSG-6 restored the production of sGAGs. Notably, the effect of TSG-6 on the synthesis of sGAGs in HNPCs was first explored in this study. According to a previous study, SGAGs, the major IVD matrix components, are one among proteoglycans that impart elastic compressive strength to IVD by water uptake [19]. Moreover, IL-1β has already been demonstrated to promote ECM degradation [20]. In this article, IL-1β stimulation obviously decreased the expression levels of collagen II and aggrecan proteins and increased the protein expression of MMP-3 in HNPCs; while over-expression of TSG-6 notably reversed the induction effect of IL-1β. Furthermore, TSG-6 could exert anti-inflammatory effects by inhibiting the MMP-1 and MMP-3 transcription and the MMP-1 activation, discovered by Guo et al. [21] from conjunctivochalasis. Collectedly, TSG-6 can inhibit NPC degeneration by ameliorating IL-1β-induced ECM degradation in NPCs.

In addition, we discovered that over-expression of TSG-6 could significantly suppress IL-1β-induced expression of TNF-α, IL-8, and IL-6 in HNPCs. Though it is unclear how TSG-6 affects the expression of the pro-inflammatory factors, a great number of studies have proved a powerful function of TSG-6 in inhibiting inflammation. Some scholars believe that TSG-6 may protect tissues from acute inflammation via direct interaction with chemokines (including CXCL8) to reduce inflammatory cells and seal pro-inflammatory cytokines [22]. In this article, TSG-6 significantly suppressed the expression levels of pain-related molecules (CGRP, NGF, SP), which is consistent with the study by Ichiseki et al. [23]. They discovered that TSG-6 over-expression could significantly inhibit the expression of pain-related molecules and chondrolytic enzymes studied in a monoiodoacetate-induced rat arthritis model.

It has been reported that the PI3K/Akt pathway mediates a great deal of cell biological activities, such as cell proliferation, senescence, and apoptosis [24]. Recent studies have proposed that activation of the PI3K/Akt pathway can effectively inhibit cell damage caused by inflammatory responses. For example, Sun et al. [25] stated that activation of the PI3K/Akt pathway significantly inhibited the inflammatory response in the treatment of chronic obstructive pulmonary disease with macrolides. Chen et al. [26] observed that TREM2 reduced neuroinflammation and neuronal apoptosis after intracerebral hemorrhage in mice by activating the PI3K/Akt pathway. As for the findings in our study, IL-1β stimulation notably inhibited activities of the PI3K/Akt pathway, while TSG-6 could significantly activate the PI3K/Akt pathway. Additionally, Lu et al. [24] also claimed that TSG-6 inhibited endoplasmic reticulum stress by regulating PI3K/AKT pathway activity. Overall, TSG-6 may reduce pain and inhibit inflammation in CDD patients by activating the PI3K/AKT signaling pathway.

## Conclusion

To sum up, TSG-6 is highly expressed in CDD patients and IL-1β-induced HNPCs. Over-expression of TSG-6 significantly ameliorates IL-1β-induced inflammatory responses, ECM degradation, and expression of pain-related molecules in HNPCs. Besides, the mechanism and role of TSG-6 in CDD patients may be achieved by activating the PI3K/AKT signaling pathway. In a word, TSG-6 serves as a potential target for CDD therapy.

## Data Availability

The datasets are available from the corresponding authors upon reasonable request.
